# Short-term effects of a nicotine-free e-cigarette compared to a traditional cigarette in smokers and non-smokers

**DOI:** 10.1186/s12890-015-0106-z

**Published:** 2015-10-12

**Authors:** Marco Ferrari, Alessandro Zanasi, Elena Nardi, Antonio Maria Morselli Labate, Piero Ceriana, Antonella Balestrino, Lara Pisani, Nadia Corcione, Stefano Nava

**Affiliations:** Department of Specialistic, Diagnostic and Experimental Medicine, Respiratory and Critical Care Unit, Alma Mater Studiorum, Sant’Orsola Malpighi Hospital, University of Bologna, Bologna, Italy; Respiratory and Critical Care Unit, Sant’Orsola Malpighi Hospital, Via Massarenti 9, 40138 Bologna, Italy; Respiratory Unit, Fondazione S.Maugeri, IRCCS, Istituto Scientifico di Pavia, Pavia, Italy; Laboratory of Biostatistics, Department of Medical and Surgical Sciences (DIMEC), Alma Mater Studiorum, University of Bologna, Bologna, Italy

**Keywords:** Electronic cigarettes, Pulmonary function tests, Smoking, Concentration of carbon monoxide in exhaled breath

## Abstract

**Background:**

A few studies have assessed the short-term effects of low-dose nicotine e-cigarettes, while data about nicotine-free e-cigarettes (NF e-cigarettes) are scanty. Concerns have been expressed about the use of NF e-cigarettes, because of the high concentrations of propylene glycol and other compounds in the e-cigarette vapor.

**Methods:**

This laboratory-based study was aimed to compare the effects of *ad libitum* use of a NF e-cigarette or and a traditional cigarette for 5 min in healthy adult smokers (*n* = 10) and non-smokers (*n* = 10).

The main outcome measures were pulmonary function tests, fraction of exhaled nitric oxide (FeNO) and fractional concentration of carbon monoxide (FeCO) in exhaled breath.

**Results:**

The traditional cigarette induced statistically significant increases in FeCO in both smokers and non-smokers, while no significant changes were observed in FeNO. In non-smokers, the traditional cigarette induced a significant decrease from baseline in FEF75 (81 % ± 35 % vs 70.2 % ± 28.2 %, *P* = 0.013), while in smokers significant decreases were observed in FEF25 (101.3 % ± 16.4 % vs 93.5 % ± 31.7 %, *P* = 0.037), FEV_1_ (102.2 % ± 9.5 % vs 98.3 % ± 10 %, *P* = 0.037) and PEF (109.5 % ± 14.6 % vs 99.2 % ± 17.5 %, *P* = 0.009). In contrast, the only statistically significant effects induced by the NF e-cigarette in smokers were reductions in FEV_1_ (102.2 % ± 9.5 % vs 99.5 ± 7.6 %, *P* = 0.041) and FEF25 (103.4 % ± 16.4 % vs 94.2 % ± 16.2 %, *P* = 0.014).

**Discussion:**

The present study demonstrated that the specific brand of NF e-cigarette utilized did not induce any majoracute effects. In contrast, several studies have shown that both traditional cigarettes and nicotine-containing e-cigarettes have acute effects on lung function. Our study expands on previous observations on the effects of NF e-cigarettes, but also for the first time describes the changes induced by smoking one traditional cigarette in a group of never smokers.

**Conclusions:**

The short-term use of the specific brand of NF e-cigarette assessed in this study had no immediate adverse effects on non-smokers and only small effects on FEV_1_ and FEF25 in smokers. The long-term health effects of NF e-cigarette use are unknown but worthy of further investigations.

**Trial registration:**

Clinicaltrials.gov: NCT02102191

## Background

Electronic cigarettes (e-cigarettes) have been proposed as a novel method for quitting smoking. The producers of e-cigarettes claim that one of the benefits of these cigarettes is that a smoker may gradually decrease the nicotine content over time, until a state of “nicotine-free” smoking is reached. This state can be reached without the smoker having to renounce “habit-automatisms” and handling, which are major obstacles to quitting smoking.

However, the recent position statement of the Forum of International Respiratory Societies [1] on the use of e-cigarettes and their potential hazards concluded that these devices should be restricted or banned until more information about their safety is available. The major concerns include the nicotine content and the potential harm due to the high concentrations of propylene glycol, which is irritant when inhaled, chemicals, such as quinoline, benzoic acid and diethylcarbonate, and other compounds found in the e-cigarette vapor.

To our knowledge there are no data on the health effects of acute use of nicotine-free e-cigarettes (NF e-cigarettes); we, therefore, designed a study to compare the changes in pulmonary function tests (PFT) and fractions of exhaled nitric oxide (FeNO) and carbon monoxide (FeCO) as a result of 5 min of *ad libitum* smoking of a NF e-cigarette or a traditional cigarette, in smokers and non-smokers.

## Methods

### Subjects

Twenty normal subjects, recruited among pulmonary fellows or attending physicians were studied: 10 were smokers (minimum of 5 pack-years) and 10 were non-smokers. Exclusion criteria were current use of any medication, the presence of any acute or chronic lung disease, neuromuscular diseases, cancer, chronic heart failure, metabolic or auto-immune diseases and acute illness during the preceding 4 weeks. Each subject was asked to sign written informed consent to the protocol approved by the Salvatore Maugeri Ethical Committee. The protocol was registered at www.clinicaltrials.gov with the number NCT02102191 on March 27, 2014.

### Protocol

Both smokers and non-smokers were randomized to smoke both the NF e-cigarette and a commercial “popular brand” standard cigarette *ad libitum* for 5 min in two different sessions according to a cross-over design (5 patients within each group smoked first the NF e-cigarette and then the commercial cigarette and 5 subjects smoked first the commercial and then the NF e-cigarette). All subjects were asked to use a similar pattern and frequency of smoke aspiration, although it cannot be assured that they did so. The subjects were also asked to refrain from smoking in the 6 h preceding the test session and not to eat or drink for at least 4 h prior to the experimental procedure.

The first smoking session started 5 min after the baseline measurement of FeCO, FeNO and PFT. The second smoking session started after a wash-out of 24 h after the end of the first session. This wash-out period was to ensure that there was no carry-over effect. The measurements of FeNO, FeCO and PFTs were repeated immediately after each smoking session.

The NF e-cigarette used in this study, ELIPS C Series (Ovale Europe S.r.l., Desenzano del Garda, Brescia, Italy), was a brand commercially available in Italy. It was formed of a steel shell with a microprocessor powered by a battery, a filter and a removable cartridge. Among the six different types of cartridge available, we chose “Natur Smoke aroma Nocciola Antistress 0 mg/mL nicotina” (Angelica, Bologna, Italy), i.e., a nicotine-free liquid with a hazelnut flavorThe liquid of the cartridge is registered by the Italian Regulatory Agency and had the following composition: glycerin >50 %, isotonic solution 5–10 %, magnesium chloride 1–5 %, natural flavor 0.1–1 %, and vitamin B12 0.1–1 %. The specific kind of NF e-cigarette chosen in the current study followed an unbiased internet search for products available and produced in Italy (e.g.Dea, Flatech, Flavour Roma). Use of the Angelica liquid was finally decided mainly due to logistic convenience since it was produced in the same city (Bologna) of investigation.

The commercial standard cigarette, Marlboro^®^ Red Label Box (Philip Morris USA Inc., Miami, FL, USA), contained nicotine 0.8 mg, carbon oxide (CO) 10 mg and tar 10 mg. According to the manufacturer [2], the components not exceeding 0.1 % of the weight of the tobacco were acetic acid 0.01, acetophenone 0.0001, ammonium hydroxide 0.3, amyl butyrate 0.0001, benzaldehyde 0.005, benzoin 0.005, benzyl alcohol 0.1, cellulose 9.3, calcium carbonate 4.6, monopotassium phosphate 1.4, potassium citrate 0.3, guar gum 0.1, and hercon70 0.1.

### Measurements

The exhaled nitric oxide (FeNO) and fractional concentration of carbon monoxide in exhaled breath (FeCO) were measured using chemiluminescense analyzers (NIOX MINO, Aerocrine AB, Solna, Sweden and Micro Smokerlyzer, Bedfont Scientific Ltd., Rochester, Kent, Great Britain, respectively) with a computerized program. The FeNO analyzer was calibrated with certified NO mixtures (100 ppb) in nitrogen.

PFT were performed with a spirometer (Chestgraph HI-105 - CHEST M.I. Inc, Tokyo, Japan). The following parameters were recorded in the sitting position: forced vital capacity (FVC), forced expiratory volume in 1 s (FEV_1_), forced expiratory flow (FEF) 25 %, 50 % and 75 % and peak expiratory flow (PEF). Spirometry was performed following the recommendations of the American Thoracic Society/European Respiratory Society (ATS/ERS) guidelines [3].

For the FeNO recording, the subjects were studied in the sitting position wearing a nose-clip and were asked to inhale as deeply as they could, to total lung capacity, while breathing through a mouthpiece and then to exhale at a flow rate of about 50 mL/s, maintaining a constant mouth pressure of 4 to 5 cm H_2_O for 10 s, aided by visual feedback on the screen of the instrument.

Each of the three different measurements (FeNO, FeCO and PFT) were separated by intervals of about 45 s.

### Statistical analysis

Data are expressed as mean ± SD or as frequencies. A Kolmogorov-Smirnov non-parametric test was applied to test the normality of the distributions. Two-way ANOVA was applied to the differences observed between basal values and those after smoking a traditional cigarette or an e-cigarette, considering smoking habit and the cross-over design as factors. The effects estimated by ANOVA are reported together with their 95 % confidence intervals (95 % CI). A two-tailed P value less than 0.05 is considered statistically significant. All analyses were performed using SPSS for Windows (ver. 21, IBM Corporation, Armonk, NY, USA).

### Sample size

Prokhorov et al. found a decrease of 2.14 % in the predicted value of FEV_1_ in 18 volunteer, regular smokers after smoking one traditional cigarette [4]. Since the standard deviation of the within-subject difference was not reported, we have estimated this value as 3.26 % (one third of the value reported as the overall standard deviation in the study by Prokhorov et al.; i.e., 9.78 %) [4]. By comparing these values versus no effect of the e-cigarette, we had to study 20 subjects in order to be able to reject the null hypothesis with a probability (power) of 0.80 and a two-sided type I error probability of 0.05. We, therefore, set the sample size as 20 subjects (10 smokers and 10 non-smokers), hypothesizing similar effects of smoking one traditional cigarette between smokers and non-smokers. The sample size was estimated by means of “PS Power and Sample Size Calculations” software (Version 3.0.43; Department of Statistics of the Vanderbilt University, Nashville, TN, USA; http://biostat.mc.vanderbilt.edu/twiki/bin/view/Main/PowerSampleSize) according to Dupont and Plummer [5].

## Results

The subjects’ characteristics are reported in Table 1.

**Table 1:** Subjects’ characteristics

	Overall	Smokers	Non-smokers	*P* value
Gender (M/F)	11/9	4/6	7/3	0.370^a^
Age (mean ± SD, years)	39.3 ± 12.6	42.3 ± 12.8	36.2 ± 12.3	0.291^b^
Weight (mean ± SD, kg)	67.9 ± 10.4	63.5 ± 10.1	72.3 ± 9.2	0.056^b^
Height (mean ± SD, cm)	169. ± 10.0	163 ± 7.4	176 ± 8.3	0.002^b^
BMI (mean ± SD, kg/m^2^)	23.5 ± 2.5	23.7 ± 3.1	23.3 ± 1.9	0.704^b^
Smoke history (pack-years) - mean ± SD (range)	-	19.4 ± 10.8 (5–35)	-	-

^a^Fisher’s exact test

^b^ *t*-test for equality of means

All the subjects completed the study protocol. A few non-smokers reported mild adverse events such as dry cough (*n* = 3) and throat irritation (*n* = 2) when smoking traditional cigarettes.

### FeCO and FeNO

**Fig. 1:**
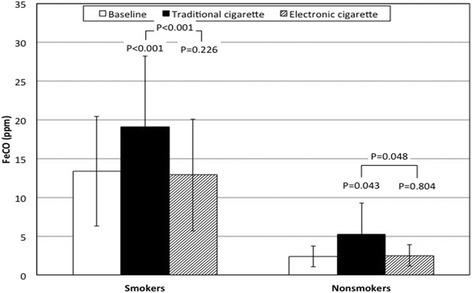
Changes in fractional concentration of carbon monoxide in exhaled breath (FeCO) in smokers and non smokers

The FeCO values in the smokers and non-smokers are shown in Figure 1. As expected, baseline FeCO values were significantly higher in smokers than in non-smokers (P < 0.001,two-way ANOVA). The signify

cance values, using the two-way ANOVA analysis, of the changes of FeCO values versus the baseline ones observed in smokers and non-smokers after smoking each type of cigarette, as well as the comparison between the traditional and e-cigarette, are also shown in Fig. 1 while the estimated effects of the two different types of cigarette in the overall population and the comparison of these effects between smokers and non-smokers, using the two-way ANOVA analysis, are presented in Table 2. In the 20 subjects studied the traditional cigarette significantly increased FeCO values (P < 0.001); this effect was significant in both groups of subjects (smokers P < 0.001; non-smokers *P* = 0.043). In contrast, the e-cigarette did not have any significant effects on FeCO (overall population *P* = 0.486; smokers *P* = 0.226; non-smokers *P* = 0.804). The increase of FeCO values observed after smoking the traditional cigarette was significantly different from the effect of the e-cigarette (overall population *P* < 0.001; smokers *P* < 0.001; non-smokers *P* = 0.048). As far as the comparison between smokers and non-smokers is concerned, no significant differences were found (traditional cigarette *P* = 0.127; e-cigarette *P* = 0.301). Likewise, the difference observed between the two types of cigarette was not significant between smokers and non-smokers (*P* = 0.067).

**Table 2:** Effects of the two different types of cigarette

	Overall population			Smokers vs. non smokers		
	Effect	95 % CI	P	Effect	95 % CI	P
FeCO (ppm)						
Traditional	4.3	(2.3 to 6.2)	<0.001	2.9	(−0.9 to 6.7)	0.127
Electronic	−0.2	(−0.8 to 0.4)	0.486	−0.6	(−1.8 to 0.6)	0.301
P value	<0.001			0.067		
FeNO (ppb)						
Traditional	−0.6	(−1.6 to 0.4)	0.219	1.4	(−0.6 to 3.4)	0.155
Electronic	−0.2	(−0.8 to 0.4)	0.512	0.8	(−0.5 to 2.1)	0.198
P value	0.372			0.501		
FEV_1_ (%)						
Traditional	−3.4	(−5.9 to −0.8)	0.013	−1.0	(−6.1 to 4.1)	0.691
Electronic	−1.7	(−4.8 to 2.9)	0.070	−2.1	(−5.7 to 1.6)	0.250
P value	0.098			0.575		
FVC (%)						
Traditional	−1.6	(−5.7 to 2.6)	0.437	−3.9	(−12.2 to 4.4)	0.331
Electronic	−1.0	(−4.8 to 2.9)	0.602	−2.2	(−9.8 to 5.4)	0.543
P value	0.584			0.448		
FEV_1_/FVC						
Traditional	−1.9	(−3.9 to 0.1)	0.065	2.0	(−2.0 to 6.1)	0.304
Electronic	−0.9	(−2.5 to 0.6)	0.226	−0.1	(−9.8 to 3.0)	0.926
P value	0.182			0.132		
PEF (%)						
Traditional	−6.5	(−11.7 to −1.3)	0.017	−7.5	(−17.8 to 2.9)	0.148
Electronic	−3.8	(−9.2 to 1.6)	0.155	−6.0	(−16.8 to 4.8)	0.254
P value	0.145			0.692		
FEF25 (%)						
Traditional	−5.8	(−10.9 to −0.6)	0.030	.4.1	(−14.1 to 6.2)	0.412
Electronic	−3.7	(−7.6 to 0.3)	0.066	−7.1	(−15.0 to 0.7)	0.073
P value	0.252			0.414		
FEF50 (%)						
Traditional	−5.0	(−9.5 to −0.4)	0.033	2.1	(−6.9 to 11.1)	0.629
Electronic	−2.9	(−6.8 to 1.1)	0.143	−2.9	(−10.8 to 5.0)	0.447
P value	0.161			0.096		
FEF75 (%)						
Traditional	−6.1	(−11.9 to −0.3)	0.040	9.3	(−2.3 to 20.9)	0.107
Electronic	−4.8	(−11.2 to 1.6)	0.132	3.6	(−16.4 to 9.3)	0.562
P value	0.657			0.037		

**Fig. 2:**
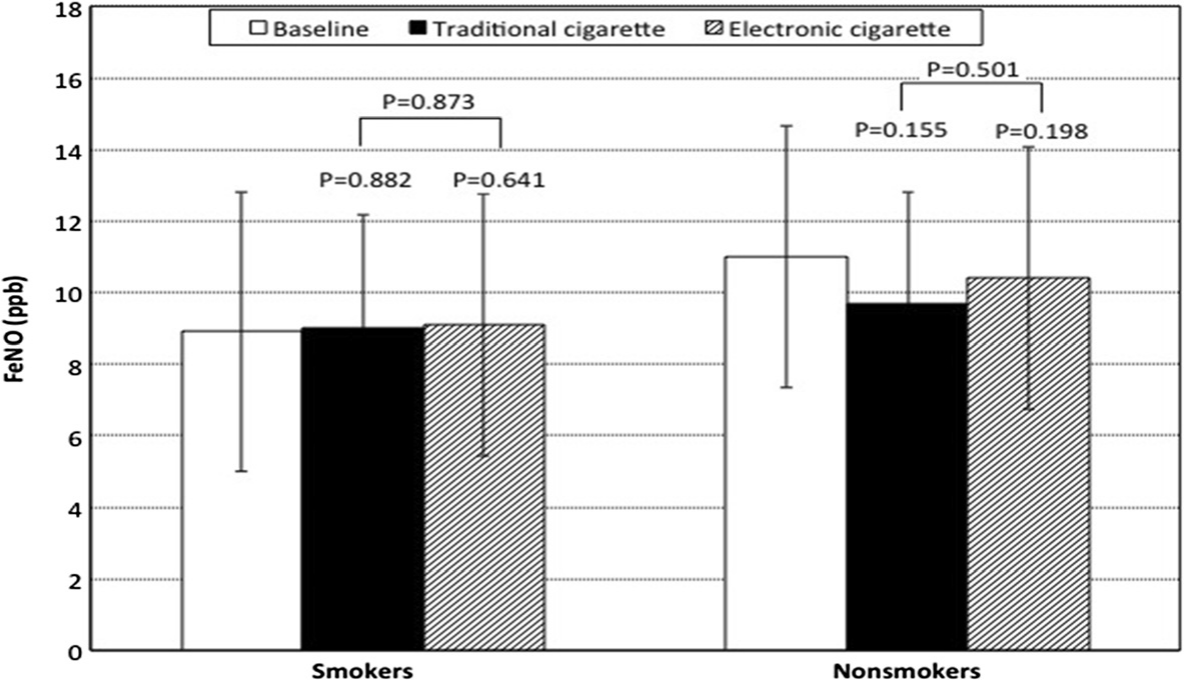
Changes in fraction of exhaled nitric oxide (FeNO) in smokers and non smokers

Baseline values of FeNO were not significantly different between smokers and non-smokers (*P* = 0.245, two-way ANOVA). No significant changes of FeNO were observed in the two groups of subjects after smoking either a traditional or e-cigarette (Fig. 2, two-way ANOVA) and no significant changes were found in the overall group of subjects studied (Table 2).

### Pulmonary function tests

All baseline pulmonary function tests (FEV_1_, FVC, FEV_1_/FVC, and PEF) were similar between smokers and non-smokers (P ≥ 0.157,Two-way ANOVA).

**Fig. 3:**
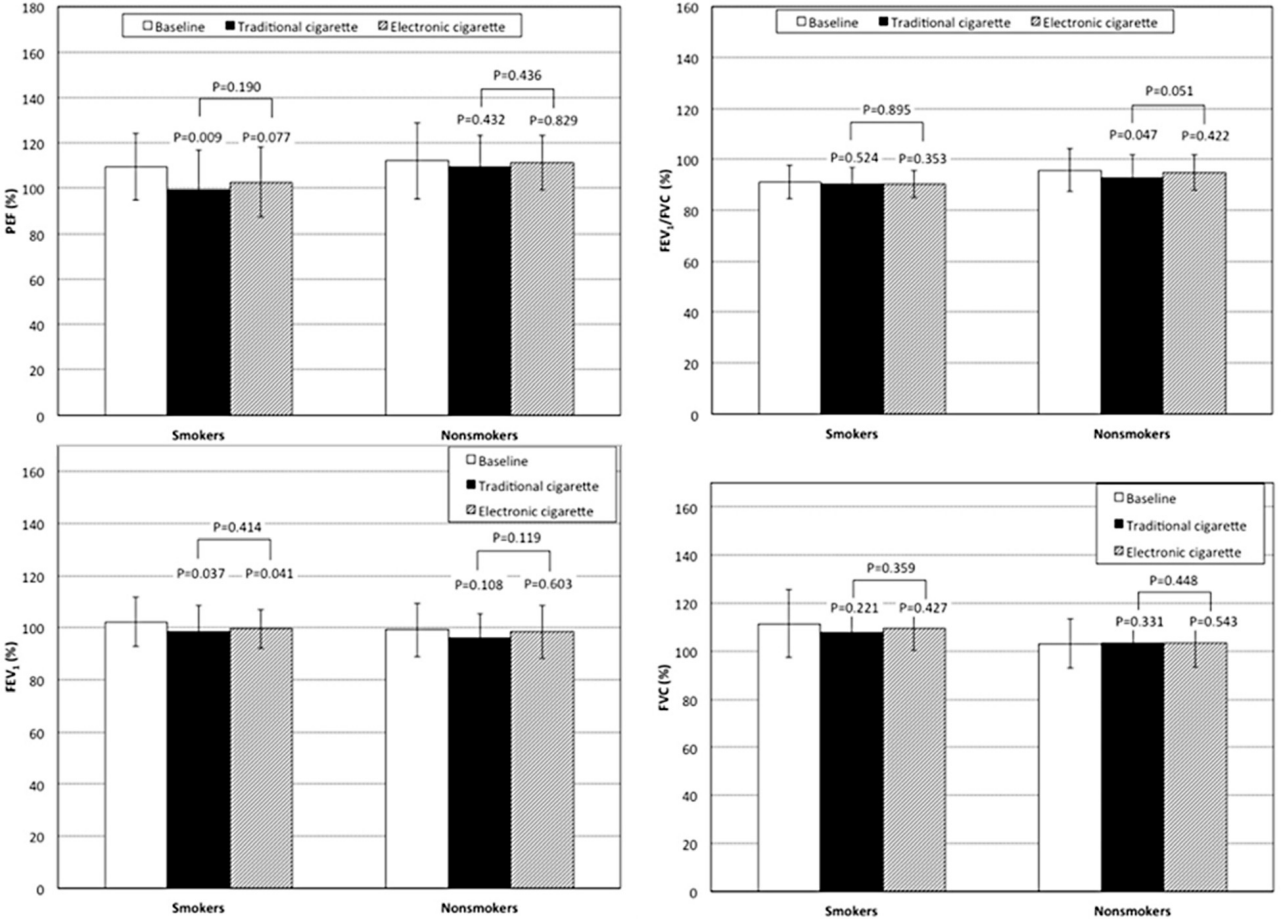
Changes in pulmonary function tests in smokers and non smokers

Smoking a traditional cigarette significantly decreased the FEV_1_/FVC in non-smokers (*P* = 0.047 two way ANOVA; Fig. 3). In addition, both types of cigarettes significantly decreased FEV_1_ values in smokers (traditional *P* = 0.037; electronic *P* = 0.041, two-way ANOVA) while the decreases in non-smokers were not significant; thus FEV1 decreased significantly in the overall population (*P* = 0.013, Two-way ANOVA) after smoking a traditional cigarette while the effect of the e-cigarette did not reach a statistically significant level (*P* = 0.070, Two-way ANOVA). Finally, the traditional cigarette significantly decreased PEF values in the overall population (*P* = 0.017, Two-way ANOVA) due to effect in the smokers (*P* = 0.009,Two-way ANOVA). The changes in FEV_1_, FVC, FEV_1_/FVC, and PEF between the two types of cigarettes were not significantly different in either smokers or non-smokers (Fig. 3) or, indeed in the overall population (Table 2).

**Fig. 4:**
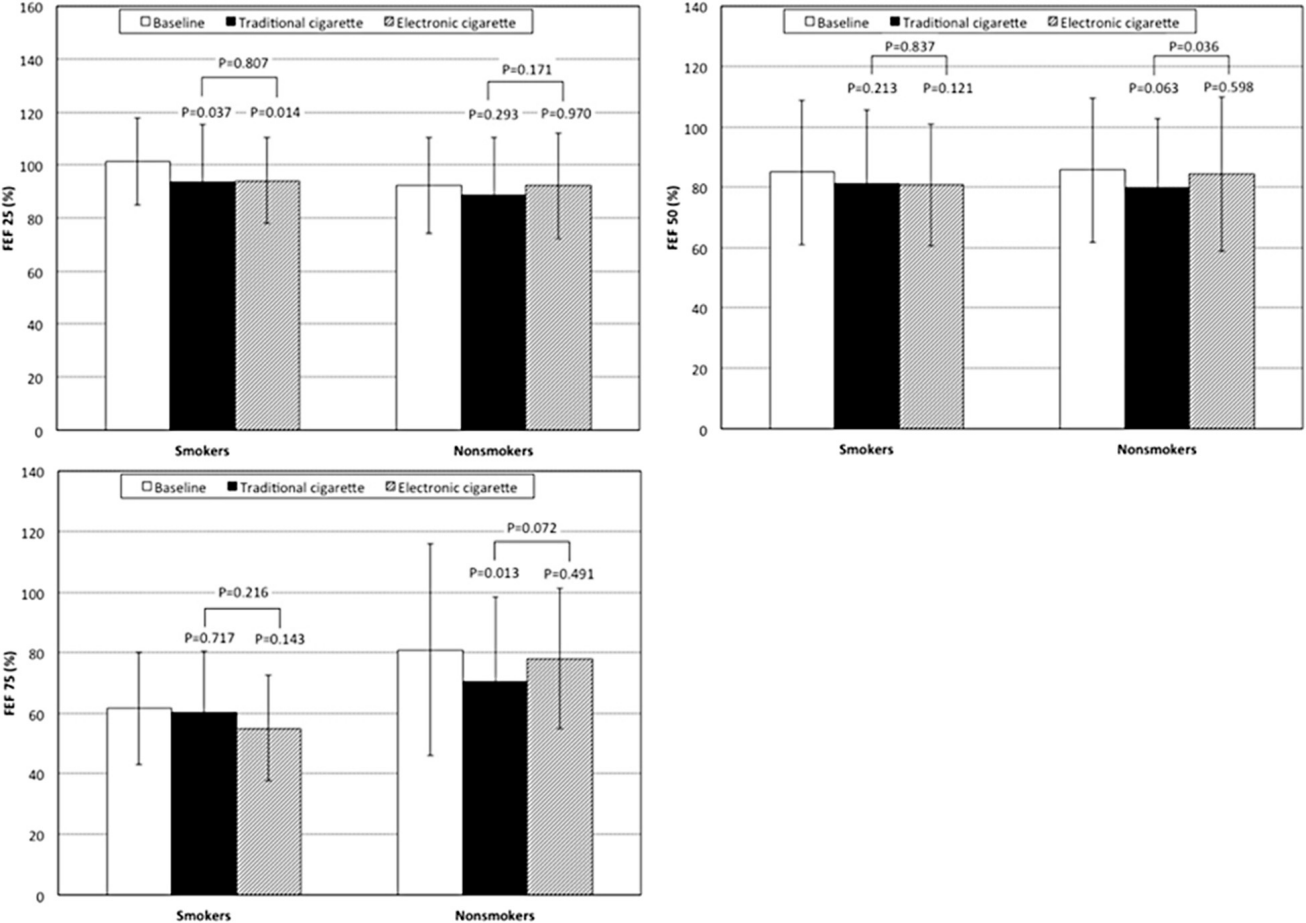
Changes in ”small airways flows” in smokers and non smokers

As far as FEF values are concerned, the traditional cigarette significantly decreased FEF25, FEF50 and FEF75 in the overall population (*P* = 0.030, *P* = 0.033, and *P* = 0.040, respectively, two-way ANOVA; Table 2), particularly due to the significant reductions of FEF25 in smokers (*P* = 0.037) and FEF75 in non-smokers (*P* = 0.013) while the reduction of FEF50 did not reach the significant levels in either smokers (*P* = 0.213) or non-smokers (*P* = 0.063) (Fig. 4). The only significant effect of the e-cigarette was a reduction of FEF25 in smokers (*P* = 0.014, two-way ANOVA). Comparing the effects of traditional and e-cigarette smoking, only a significantly greater reduction of FEF50 was found after traditional cigarette smoking in non-smokers (*P* = 0.036, two-way ANOVA).

As far as concerns the comparison between smokers and non-smokers, higher values of FEF75 were found after smoking an e-cigarette than after smoking a traditional cigarette, whereas the inverse was the case in smokers (*P* = 0.037, two-way ANOVA) (Table 2, Fig. 4c).

## Discussion

We found that the specific brand of NF e-cigarettes used in this study was not associated with major acute physiological changes, causing only small, albeit statistically significant decreases in FEF25 and FEV_1_ in the group of smokers. In contrast, smoking a traditional cigarette induced immediate bronchoconstriction in non-smokers.

Tobacco cigarettes are one of the most important risk factors for disease worldwide and the primary goal of tobacco control is to reduce the mortality and morbidity associated with its use.

E-cigarettes have gained popularity in the last few years, mainly because of the advertisements of their producers, who claim that smoking tar-free cigarettes is associated with reduced risk for the health.

A recent systematic review assessing the efficacy of e-cigarettes included six experimental studies and six cohort studies. The authors concluded that the use of e-cigarettes can reduce the number of cigarettes smoked and withdrawal symptoms, but that it was associated with some adverse events, such as mouth and throat irritation, nausea, headache and dry cough [6].

E-cigarettes have been shown to induce immediate adverse physiological effects after short-term use, similar to those observed with tobacco smoking [7]. The statement of the Forum of International Respiratory Societies [1] on the use of e-cigarettes and their potential hazards concluded that, considering the above-mentioned facts, the use of these devices should be restricted until more information on their safety becomes available.

More recently, NF e-cigarettes have been released on the market with the aim of minimizing the adverse events linked with the use of nicotine-delivering e-cigarettes.

One of the concerns of using NF e-cigarettes is that the devices contain high concentrations of glycol, which is a known irritant when inhaled. Other potentially dangerous ingredients that may be found in NF e-cigarettes are solvents, genotoxins and various other chemicals and animal carcinogens (e.g., benzoic acid, quinoline).

The act of ‘smoking’ an e-cigarette is called ‘vaping’ and it mimics smoking so, in addition to delivering nicotine, it can address both pharmacological and behavioral components of cigarette addiction. Indeed, the notable reduction in craving achieved by NF e-cigarettes demonstrates the ability of physical stimuli to suppress cravings independently of the administration of nicotine [8–13].

In a randomized, controlled trial of smoking cessation aided by nicotine e-cigarettes, NF e-cigarettes or nicotine patches performed in 657 people, Bullen et al. [13] found a rather disappointing percentage of abstinence at 6 months and the modest overall effect of the three methods (nicotine e-cigarettes, NF e-cigarettes, nicotine patches) did not allow a demonstration of the superiority of nicotine e-cigarettes over NF e-cigarettes.

The present study demonstrated that the specific brand of NF e-cigarette utilized did not induce any major acute effects. In contrast, several studies have shown that both traditional cigarettes and nicotine-containing e-cigarettes have acute effects on lung function: Vardavas et al. [7] showed that e-cigarettes containing a dose of 11 mg of nicotine significantly increased the impedance and resistance after 5 min of smoking, while Flouris et al. [12] demonstrated a significant immediate decrease in lung function only when smoking traditional cigarettes and not with e-cigarettes. Similar data were also reported by Unverdorben et al. [14], who showed a significantly greater decrease in specific airway conductance and FEF25 after smoking a conventional cigarette than after a low nicotine (5 mg) e-cigarette.

Our study expands on previous observations on the effects of NF e-cigarettes, but also for the first time describes the changes induced by smoking one traditional cigarette in a group of never smokers. Interestingly in these subjects the decreases in PFT values were much more pronounced (although not-significantly) than in the smokers, possibly because the airways of the former were more ‘naïve’ to noxious stimuli, which may have induced greater narrowing of the lumen of the peripheral airways, due to localized edema or smooth muscle contraction. The change in pulmonary function of the smokers was less pronounced than that reported in some previous studies and did not achieve statistical significance, despite the rate of change being quite similar to the above mentioned investigations. This may be due to the less sophisticated method of assessing airway narrowing in our study than in the study by Vardavas et al. (PTF vs impulse oscillometry, respectively) [7] or the longer and more intense habit of smoking of our group of subjects, which could have minimized the response.

The specific brand of NF e-cigarettes used in the present study did not induce any significant changes in smokers apart from decreases in FEV_1_ and FEF25. This latter finding is not easy to explain, and there are no investigations so far that have assessed the potential role of components of NF e-cigarettes on airway reactivity. Although both groups of subjects inhaled effectively - as illustrated by the significant increase in FeCO - only the group of active smokers reached levels of CO suggestive of deep inhalation (about 20 ppm), while the non-smokers had lower values (about 5 ppm). The higher levels of FeCO observed in smokers than in non-smokers may be explained by the previous CO exposure from tobacco smoking in the former. Indeed, according to the study protocol, smokers should have refrained from smoking in the 6 h preceding the experiments while the half-life of expired CO is about 4 h, depending on exercise.

As expected, the FeNO level was reduced in the smokers, but no significant changes were observed in either group after cigarettes smoking. This is in contrast to what was observed in several studies [15–18], but not with the data of Chambers et al. [19] who found an increase in the level of exhaled NO minutes after smoking a traditional cigarette. Balint et al. [20] also found no significant change in the concentration of exhaled NO after smoking two cigarettes, but the concentrations of NO metabolites (NO_2_^−^ + NO_3_^−^) were significantly increased. These findings suggest that NO might be trapped at the epithelial surface of airways in the formation of bioequivalent oxides of nitrogen such as peroxynitrite and S-nitrosothio.

The present study has some limitations that need to be discussed. First, the technique used to detect airway narrowing may not be as sensitive as the impulse oscillatory technique and it has been shown that changes in flow resistance usually precede variations in PFT. Indeed we assessed only crude spirometry, while DLCO and lung volumes and perhaps measurements of airway reactivity and particulate/vapor burden, may have given more insights into the problem. The *ad libitum* smoking of the cigarette may also be criticized because of the lack of standardization between subjects and because it is likely that a current smoker would smoke more than a non–smoker. The sample size (20 subjects) may be considered quite small, although it was based on a sophisticated calculation involving previously reported data (4,5), so we are confident that the data collected may be representative enough of the changes induced by smoking either NF e-cigarettes or traditional ones.

## Conclusions

In conclusion, smoking the NF e-cigarette studied in the present investigation had no immediate adverse effects after short-term use in non-smokers and a small effect on FEV_1_ and FEF25 in smokers. In contrast, acute traditional cigarette smoking was associated with more detrimental effects on PFT in non-smokers than in smokers, although differences were not statistically significant. The long-term health effects of NF e-cigarette use are unknown but worthy of further investigation.

## Abbreviations

(NF e-cigarettes): Nicotine-free e-cigarettes
(FeNO): Fractional exhaled nitric oxide
(FeCO): Fractional concentration of carbon monoxide
(PTF): Pulmonary function tests
